# Predictive markers of transmission in areas with different malaria endemicity in north-eastern Tanzania based on seroprevalence of antibodies against *Plasmodium falciparum*

**DOI:** 10.1186/s13104-021-05818-y

**Published:** 2021-10-30

**Authors:** Robert D. Kaaya, Debora C. Kajeguka, Johnson J. Matowo, Arnold J. Ndaro, Franklin W. Mosha, Jaffu O. Chilongola, Reginald A. Kavishe

**Affiliations:** 1 Departmentof Parasitology and Entomology, Faculty of Medicine, Kilimanjaro Christian Medical University College, Moshi, Tanzania; 2 Pan-African Malaria Vector Research Consortium, Moshi, Tanzania; 3 Department of Microbiology, Faculty of Medicine, Kilimanjaro Christian Medical University College, Moshi, Tanzania; 4 Kilimanjaro Christian Medical Centre (KCMC), Moshi, Tanzania; 5 Department of Biochemistry and Molecular Biology, Faculty of Medicine, Kilimanjaro Christian Medical University College, Moshi, Tanzania

**Keywords:** Malaria, *Plasmodium falciparum*, Seroprevalence, Transmission, Tanzania

## Abstract

**Objective:**

A community-based cross-sectional study was done to assess *Plasmodium falciparum* exposure in areas with different malaria endemicity in north-eastern Tanzania using serological markers; *PfAMA-1* and *PfMSP-1*_*19*_.

**Results:**

Bondo had a higher seroprevalence 36.6% (188) for *PfAMA-1* as compared to Hai 13.8% (33), χ^2^ = 34.66, p < 0.01. Likewise, Bondo had a higher seroprevalence 201(36.6%) for *PfMSP-1* as compared to Hai 41 (17.2%), χ^2^ = 29.62, p < 0.01. Anti-*PfAMA-1* titters were higher in malaria positive individuals (n = 47) than in malaria negative individuals (n = 741) (p = 0.07). Anti-*PfMSP*-1 antibody concentrations were significantly higher in malaria-positive individuals (n = 47) than in malaria-negative individuals (n = 741) (p = 0.003). Antibody response against *PfAMA-1* was significantly different between the three age groups; < 5 years, 5 to 15 years and > 15 years in both sites of Bondo and Hai. Likewise, antibody response against *PfMSP-1*_*19*_ was significantly different between the three age groups in the two sites (p < 0.001). We also found significant differences in the anti-*PfAMA-1*and anti-*PfMSP-1*_*19*_ antibody concentrations among the three age groups in the two sites (p = 0.004 and 0.005) respectively. Immunological indicators of *P. falciparum* exposure have proven to be useful in explaining long-term changes in the transmission dynamics, especially in low transmission settings.

**Supplementary Information:**

The online version contains supplementary material available at 10.1186/s13104-021-05818-y.

## Introduction

Africa carries the highest burden of malaria with more than 70% of all malaria cases and deaths [1]. Each year, 10 to 12 million people contract malaria and more than 80,000 dies [2, 3]. *Plasmodium falciparum* is mainly responsible for 99.7% of estimated malaria cases [4].

In many countries, local malaria transmission has decreased due to the extensive efforts being devoted to malaria control and elimination [5]. *P. falciparum* accounts for 96 percent of cases [6], malaria prevalence varies from < 1 percent in the highlands of Arusha to as high as 15 percent in the Southern Zone and 24 percent along the Lake and Western Zones. Immunity to *P. falciparum* malaria is poorly understood, however, evidence shows that antibody-dependent cellular mechanisms play a key role in immunity against *P. falciparum* malaria parasite [7, 8]. The rate of its development is believed to be associated with transmission intensity which is stage-specific and is rarely sterile [6]. In many epidemiological studies, the determination of malaria transmission has been based on the antibody levels against *P. falciparum* antigens [9]. Recent immunological studies revealed that antibodies against merozoite antigens act as biomarkers of malaria exposure and that, with increasing exposure and responses of higher levels, antibodies may act as biomarkers of protective immunity [10].

Apical membrane antigen 1 (AMA-1) is expressed on merozoites and sporozoites of *P. falciparum* as a type I integral membrane protein [11] while Merozoite surface protein1 (MSP-1), is a highly conserved protein among *Plasmodium* species as well as the most abundant protein expressed on the surface of merozoites [12]*.* Antibodies against MSP-1 and AMA-1 antigens are potential markers of both exposure to *P. falciparum* and protection against the disease [7, 13] and have proven to be informative, in areas where transmission has dropped to low sustained levels, for monitoring the timing and magnitude of transmission reduction [13] as well as in obtaining epidemiological information in malaria control programmes [14].

In areas with low malaria transmission, it has become extremely difficult to detect changes in transmission intensity using conventional methods such as the entomologic inoculation rate (EIR) or malaria prevalence rates. Low transmission areas (low endemicity) sometimes have low mosquito density, below the detection limits of common mosquito trapping methods [15, 16] and the parasite prevalence also becomes less reliable [17–19]. Malaria serological markers may aid in estimating malaria transmission intensity [20–22]. Seroconversion rates may provide insight into recent changes in malaria transmission [23]. Due to the fact antibodies can persist for months or years after infection, seroconversion rates are less affected by the effects of unstable or seasonal transmission [20, 21]. We investigated the antibody response to recombinant AMA-1 and MSP-1 in individuals living in two regionally distinct malaria-endemic zones.

## Main text

### Materials and methods

#### Study area

The study was conducted during April and December 2014 in two different areas of the Tanzanian mainland. The first site was Bondo in the Tanga region, inhabited by 7970 people [24]. The second study site was Hai in Kilimanjaro region [14]. Participant recruitment procedures and study design have been previously described [25], (Additional file 1: Fig. S1).

#### Participant enrolment and sample collection

Participants 2 years of age and above who reside in the study areas were enrolled in the study. A blood sample was obtained by finger prick, a portion of blood was used for malaria rapid test, which was performed on-site. A blood spot was prepared for each participant, then dried and stored for further analysis. A 3.0 mm diameter circle of dried blood spot (equivalent to 2 µl whole blood/1 µl serum) was reconstituted in 200 µl of sodium azide-phosphate buffered saline-tween (0.05%) (PBST/0.1% Azide).

#### Enzyme-linked immuno-sorbent assay (ELISA)

Indirect immunosorbent Assay (ELISA)was performed using two *P. falciparum* surface antigens, *P. falciparum MSP 1*_*19*_ (Pf*MSP 1*_*19*_) and *P. falciparum AMA-1*(*PfAMA-1*) [21].

#### Malaria parasite detection by polymerase chain reaction (PCR)

Parasite DNA was extracted using the simple Chelex–Saponin method, *Plasmodium* nucleic acid amplification was conducted using genus-specific reverse and forward primers (rPLU6-5′TTAAAATTGTTGCAGTTAAAACG3′ and rPLU5-5′CCTGTTGTTGCCTTAAACTCC3′) targeting small sub-unit ribosomal RNA (ssurRNA) of the parasite. A reaction mix of 20 µl per sample was used, 5 µl of template DNA extracted from participants whole blood plus 15 µl of nuclease-free water, dNTPs, Taq enzyme, buffers and salts. Amplification conditions were, 95 °C for 5 min followed by 30 cycles of 94 °C for 1 min, 58 °C for 2 min and 72 °C for 5 min then one final extension cycle at 72 °C for 10 min. Amplification products were run in Ethidium bromide agarose gel (2%) electrophoresis at 120 V, 50 watts and 120 mA. The amplified bands were visualized under ultra-violet light trans-illuminator [26, 27].

#### Data analysis

All data were analyzed using SPSS 20.0 software (SPSS Inc., Chicago, IL, USA) and GraphPad Prism8 software (San Diego, CA). After verifying that Optical density (OD) values were not normally distributed (p < 0.0001; Anderson–Darling test), non-parametric tests were performed to compare the OD. The Mann–Whitney test was used for the comparison of Antibody levels of two independent groups. The non-parametric Kruskal–Wallis test was used for the comparison of more than two groups. Pearson’s Chi-squared (χ^2^) test was used to compare two proportions.

### Results

#### Population characteristics and malaria prevalence

The study enrolled a total of 788 participants, 239 (30.3%) from Hai and 549 (69.7%) from Bondo. Males were 283 (35.9%) and females were 505 (64.1%). About 405 (51.4%) participants had more than 15 years of age, 212 (26.9%) were between 5 and 15 years and 171 (21.7%) were below 5 years. The malaria prevalence by mRDT was 8.6% (47) in Bondo and 0% in Hai (Fisher exact test *p < 0.001). By PCR, malaria prevalence was 20.4% (161), with Bondo having a higher prevalence 28.1% (n = 154) than Hai 2.9%, (n = 7), χ^2^ = 64.64, p < 0.01 (Additional file 2: Table S1).

#### Seroprevalence of anti-*PfAMA-1* and *PfMSP-1*_*19*_ antibodies

Bondo had a higher seroprevalence 36.6% (188) for *PfAMA-1* as compared to Hai 13.8% (33), χ^2^ = 34.66, p < 0.01. Likewise, Bondo had a higher seroprevalence 201(36.6%) for *PfMSP-1* as compared to Hai 41 (17.2%) (χ^2^ = 29.62, p < 0.01). In Bondo, participants with more than 15 years had a significantly higher seroprevalence of *PfAMA-1* 61.7% (116) (χ^2^ = 58.69, p < 0.001) and *PfMSP-1*_*19*_ 63.7 (128) (χ^2^ = 65.36, p < 0.001) as compared to other age groups. Likewise, participants with 5–15 years and < 5 years had a higher prevalence of malaria as measured by mDRT (χ^2^ = 30.76, p < 0.001) (Table 1).

**Table 1:** Age-specific prevalence of malaria by serology, mRDT, microscopy and PCR

Study site	Age group	PfAMA-1 % (n)		PfMSP-1_19_ % (n)		mRDT % (n)		PCR % (n)	
		Positive	Negative	Positive	Negative	Positive	Negative	Positive	Negative
Bondo	**< **5 years	9.0 (17)	32.7 (118)	14.4 (29)	30.5 (106)	46.8 (22)	22.5 (113)	18.8 (29)	26.8 (106)
	5–15 years	29.3 (55)	36.6 (132)	21.9 (44)	41.1 (143)	48.9 (23)	32.7 (164)	36.4 (56)	33.2 (131)
	> 15 years	61.7 (116)	30.7 (111)	63.7 (128)	28.4 (99)	4.3 (2)	44.8 (225)	44.8 (69)	40.0 (158)
		χ^2^ = 58.69, p < 0.001		χ^2^ = 65.36, p < 0.001		χ^2^ = 30.76, p < 0.001		χ^2^ = 3.8, p = 0.1	
Hai	**< **5 years	18.2 (6)	14.6 (30)	9.8 (4)	16.2 (32)	0.0 (0)	15.1 (36)	14.3 (1)	15.1 (35)
	5–15 years	6.1 (2)	11.2 (23)	4.9 (2)	11.6 (23)	0.0 (0)	10.5 (25)	28.6 (2)	9.9 (23)
	> 15 years	75.8 (25)	74.3 (153)	85.4 (35)	72.2 (143)	0.0 (0)	74.5 (178)	57.1 (4)	75.0 (174)
		*p = 0.6		*p = 0.2		–		*p = 0.2	

*Computed by Fisher exact test

#### Anti-*PfAMA-1* and *PfMSP-1*_*19*_ antibody concentrations

Anti-*PfAMA-1* titters were higher in malaria positive individuals (n = 47) than in malaria negative individuals (n = 741) (Mann–Whitney U test, p = 0.07) (Additional file 3: Fig. S2A). Anti-*PfMSP*-1 antibody concentrations were significantly higher in malaria-positive individuals (n = 47) than in malaria-negative individuals (n = 741) (Mann–Whitney U test, p = 0.003) (Additional file 3: Fig. S2B).

**Fig. 1:**
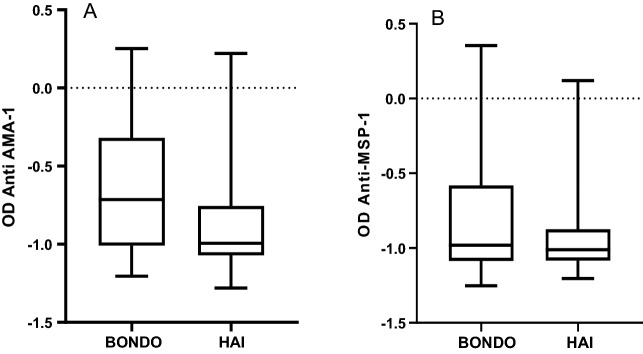
A graph showing mean OD values for *PfAMA-1* (**A**) and *PfMSP-1*_*19*_ (**B**) at Bondo and Hai sites. Presented in the Y-axis is the Log_10_ transformed OD values in two sites (X-axis)

We determined whether the two sites differed in antibody concentration and found that anti-*PfAMA-1* antibody concentrations, were higher among participants in Bondo (n = 549) as compared in Hai (n = 239), (Mann–Whitney U test, p < 0.001) (Fig. 1A). Anti-*PfMSP-1* antibody concentrations were higher among participants in Bondo (n = 549) than those of Hai (n = 239), (Mann–Whitney U test, p = 0.01) (Fig. 1B).

**Fig. 2:**
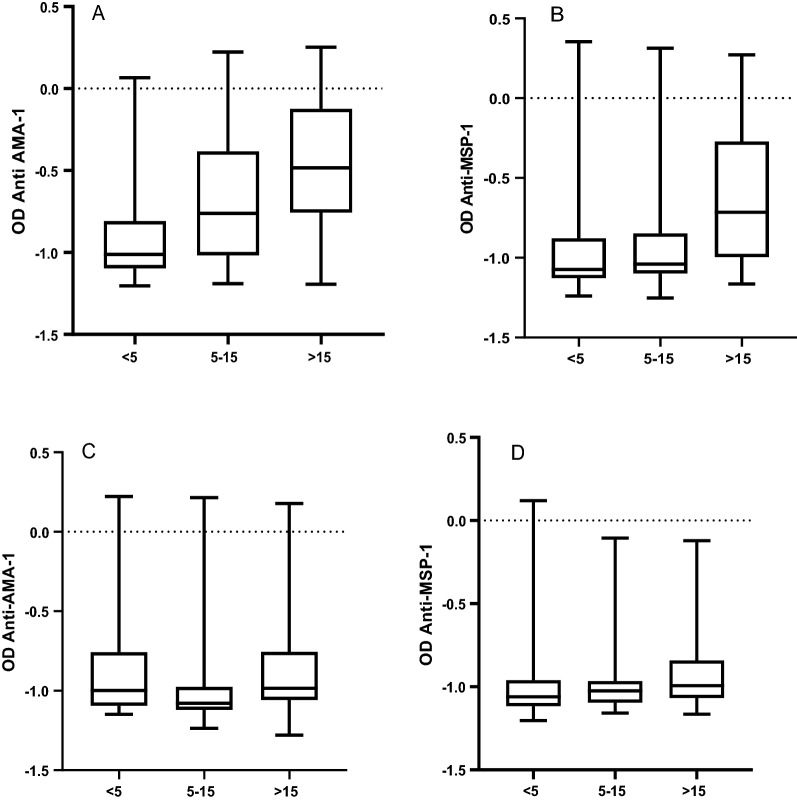
A graph showing mean OD values for anti-*PfAMA-1* antibodies and anti-*PfMSP-1*_*19*_ antibodies. **A**, **B** Show OD values for anti-*PfAMA-1* and anti-*PfMSP-1*_*19*_ in Bondo respectively. **C**, **D** Show OD values for anti-*PfAMA-1* ad anti-*PfMSP-1*_*19*_ antibodies in Hai respectively. Presented in the Y-axis is the log_10_ transformed mean OD values in different age groups (X-axis)

In assessing whether these differences were influenced by age, we calculated the differences among < 5 years, 5 to 15 years and > 15 years per site. Antibody response against *PfAMA-1* was significantly different between the three age groups in both sites. (Kruskal–Wallis test, p < 0.001) (Table 1). Likewise, antibody response against *PfMSP-1*_*19*_ was significantly different between the three-age groups in the two sites (Kruskal–Wallis test, p < 0.001) (Table 1). We also found significant differences in the anti-*PfAMA-1*antibody concentrations among the groups (Kruskal–Wallis test, p = 0.004), as indicated in Fig. 2A, B. Lastly, we also noted significant differences in the anti-*PfMSP-1*_*19*_ antibody concentrations among the age groups (Kruskal–Wallis test, p = 0.005) (Fig. 2C, D).

## Discussion

The purpose of this study was to use immunological markers to investigate malaria transmission patterns in areas with diverse malaria endemicities.

In this study, malaria prevalence by PCR in Bondo was 28.1%. Since Bondo is a malaria-endemic area, malaria transmission occurs nearly all year long with a peak period from April to June. No significant difference was observed in malaria prevalence among age groups in the present study. This is contrary to the study conducted in 2011 which suggested a widening of the age group at risk for malaria infection to older children of 5–15 years [28]. A previous study conducted in two villages in the same region about 70 km from the current study found a re-emergence of malaria despite previous reports of a decline in malaria [29]. It is estimated that parasite prevalence at that time was 25% and it stayed there throughout 2016 [30]. Hai site had a very low malaria prevalence, thus remaining an area of low transmission and The mRDT tests did not detect any active infections, which suggests low-density parasite circulating in the population, similar to earlier findings [31]. There is, however, some evidence that individuals harbouring sub-microscopic parasites could be sources of new infections since mosquitoes can carry parasites with very low density (< 5 parasites/µl) [26, 32, 33], and hence, the use of a more sensitive diagnostic tool like PCR in clinical malaria diagnosis is necessary. Consequently, scientific evidence from these findings is consistent with the notion of mass drug therapy for individuals with microscopic parasites considering efforts to eliminate malaria.

In our study, Interestingly, when the age-dependent analysis was done, older children (5–15 years) had a relatively low seroprevalence to *PfAMA-1* antigens as compared to younger children and Adults. Antibodies to malaria antigens can explain long-term changes in malaria transmission dynamics [21]. In 2009 a survey conducted in Moshi district, an area bordering Hai found a low seroprevalence in younger children suggesting very low exposure to malaria parasites [34]. In populations with low immunity, such as young children, antibodies to MSP-1 act as a significant biomarker of malaria exposure and with increasing exposure the antibodies may contribute to protective immunity [10].

Seroprevalence in moderate malaria transmission setting such as Bondo can play a small role in determining malaria transmission patterns although seroprevalence is almost two folds higher than Hai. A slight decline in seroprevalence was observed in the study area when compared with previous studies [21, 31], indicating a long-term reduction in malaria parasite exposure**,** which may be attributed to intense malaria interventions in Tanzania [35, 36].

Study results showed that the overall concentration of *PfMSP-1*_*19*_ was significantly higher in participants with positive malaria tests than in non-positive participants. As expected Bondo had higher antibody concentrations against both antigens as compared to Hai. Children with < 5 years present with low antibody titters suggesting a lack of recent malaria exposure and this makes the group vulnerable to the symptomatic manifestation of the disease. Earlier findings revealed that more than half of the participants reported being symptomatic and were malaria positive by mRDT [21]. There is evidence of malaria transmission in low malaria-endemic areas, where traditional malaria indicators like prevalence and sporozoite levels may underestimate the burden of the disease.

### Conclusion

In this study, immunological markers were found to be a useful indicator of ongoing malaria transmission, especially in low-endemic areas. Routine malaria surveillance can be made more effective by using these immunological markers to highlight the importance of customized and targeted control interventions.

## Study limitation

This study might not explain the recent changes in malaria transmission since it was a cross-sectional survey. A longitudinal study would have been appropriate in explaining seasonal variations in malaria infection rates across the study areas.

## Supplementary Information

**Additional file 1: Figure S1.** Map of Tanzania showing the study sites, the map was produced using ArcGIS version 10.3 software.

**Additional file 2: Table S1.** Prevalence of Malaria by serology, mRDT, Microscopy and PCR.

**Additional file 3: Figure S2.** A graph showing mean OD values for *PfAMA-1* (A) and *PfMSP-1*_*19*_ (B) among malaria positive and negative individuals. Presented in the Y-axis is the Log_10_ transformed OD values among malaria positives and negatives (X-axis).

## Data Availability

All data generated or analysed during this study are included in this published article.

## Abbreviations

CRERC: College Research and Ethics Review Committee
OD: Optical density
AMA-1: Apical membrane antigen1
PCR: Polymerase chain reaction
ELISA: Enzyme-Linked Immuno-Sorbent Assay
ssurRNA: Small sub-unit ribosomal RNA
*PfMSP 1*_*19*_: *Plasmodium falciparum Merozoite Surface Protein 1*
*PfAMA-1*: *Plasmodium falciparum Apical Membrane Antigen 1*

## References

[1] WHO. World Malaria Report-2019. Vol. WHO/HTM/GM, World Health Organisation. 2019. p. 238. https://www.who.int/malaria/publications/world-malaria-report-2019/World-Malaria-Report-2019-briefing-kit-eng.pdf?ua=1. Accessed 20 May 2020.

[2] MalariaSpot. Malaria in Tanzania. MalariaSpot. 2016. https://malariaspot.org/en/eduspot/malaria-in-tanzania/. Accessed 25 May 2020.

[3] Kilonzi M, Minzi O, Mutagonda R, Sasi P, Kamuhabwa A, Aklillu E (2019). Comparison of malaria treatment outcome of generic and innovator’s anti-malarial drugs containing artemether–lumefantrine combination in the management of uncomplicated malaria amongst Tanzanian children. Malar J.

[4] WHO. Malaria Fact Sheet. World Health Organisation. 2020. https://www.who.int/news-room/fact-sheets/detail/malaria. Accessed 20 May 2020.

[5] Dhiman S (2019). Are malaria elimination efforts on right track? An analysis of gains achieved and challenges ahead. Infect Dis Poverty.

[6] MoHCDGEC (Tanzania Mainland), MoH (Zanzibar), NBS, OCGS, and ICF.2017. Tanzania Malaria Indicator Survey (TMIS) 2017. Dar es Salaam, Tanzania, and Rockville, Maryland, USA

[7] Greenhouse B, Ho B, Hubbard A, Njama-Meya D, Narum DL, Lanar DE (2011). Antibodies to *Plasmodium falciparum* antigens predict a higher risk of malaria but protection from symptoms once parasitemic. J Infect Dis.

[8] Sabchareon A, Burnouf T, Ouattara D, Attanath P, Bouharoun-Tayoun H, Chantavanich P (1991). Parasitologic and clinical human response to immunoglobulin administration in falciparum malaria. Am J Trop Med Hyg.

[9] Roy A, Adak T (2005). Evaluation of malaria endemicity by peptide ELISA. J Commun Dis.

[10] Stanisic DI, Fowkes FJI, Koinari M, Javati S, Lin E, Kiniboro B (2015). Acquisition of antibodies against *Plasmodium falciparum* merozoites and malaria immunity in young children and the influence of age, force of infection, and magnitude of response. Infect Immun.

[11] Yap A, Azevedo MF, Gilson PR, Weiss GE, O’Neill MT, Wilson DW (2014). Conditional expression of apical membrane antigen 1 in *Plasmodium falciparum* shows it is required for erythrocyte invasion by merozoites. Cell Microbiol.

[12] Kale S, Yadav CP, Rao PN, Shalini S, Eapen A, Srivasatava HC (2019). Antibody responses within two leading *Plasmodium vivax* vaccine candidate antigens in three geographically diverse malaria-endemic regions of India. Malar J.

[13] Wong J, Hamel MJ, Drakeley CJ, Kariuki S, Shi YP, Lal AA (2014). Serological markers for monitoring historical changes in malaria transmission intensity in a highly endemic region of Western Kenya, 1994–2009. Malar J.

[14] Kerkhof K, Sluydts V, Willen L, Kim S, Canier L, Heng S (2016). Serological markers to measure recent changes in malaria at population level in Cambodia. Malar J.

[15] Oesterholt MJ, Bousema JT, Mwerinde OK, Harris C, Lushino P, Masokoto A (2006). Spatial and temporal variation in malaria transmission in a low endemicity area in northern Tanzania. Malar J.

[16] Smith T, Charlwood JD, Takken W, Tanner M, Spiegelhalter DJ (1995). Mapping the densities of malaria vectors within a single village. Acta Trop.

[17] Hay SI, Smith DL, Snow RW (2008). Measuring malaria endemicity from intense to interrupted transmission. Lancet Infect Dis.

[18] Beier JC, Killeen GF, Githure JI (1999). Short report: Entomologic inoculation rates and *Plasmodium falciparum* malaria prevalence in Africa. Am J Trop Med Hyg.

[19] Yekutiel P (1960). Problems of epidemiology in malaria eradication. Bull World Health Organ.

[20] Coran P, Coleman P, Riley E, Drakeley C (2007). Serology: a robust indicator of malaria transmission intensity?. Trends Parasitol.

[21] Drakeley CJ, Corran PH, Coleman PG, Tongren JE, McDonald SL, Carneiro I (2005). Estimating medium- and long-term trends in malaria transmission by using serological markers of malaria exposure. Proc Natl Acad Sci USA.

[22] Mwanziva C, Shekalaghe S, Ndaro A, Mengerink B, Megiroo S, Mosha F (2008). Overuse of artemisinin-combination therapy in Mto wa Mbu (river of mosquitoes), an area misinterpreted as high endemic for malaria. Malar J.

[23] Stewart L, Gosling R, Griffin J, Gesase S, Campo J, Hashim R (2009). Rapid assessment of malaria transmission using age-specific seroconversion rates. PLoS ONE.

[24] PHC. Population and Housing Census-Tanzania (2012). National Bureau Of Statistics: United Republic of Tanzania. 2013. www.nbs.go.tz/. Accessed 20 June 2018.

[25] Kajeguka DC, Kaaya RD, Mwakalinga S, Ndossi R, Ndaro A, Chilongola JO (2016). Prevalence of dengue and chikungunya virus infections in north-eastern Tanzania: a cross sectional study among participants presenting with malaria-like symptoms. BMC Infect Dis.

[26] Miguel RB, Coura JR, Samudio F, Suárez-Mutis MC (2013). Evaluation of three different DNA extraction methods from blood sample colected in dried blood filter papers in Plasmodium subpatent infection from the Amazon Region Brazil. Rev Inst Med Trop Sao Paulo.

[27] Haanshuus CG, Mohn SC, Mørch K, Langeland N, Blomberg B, Hanevik K (2013). A novel, single-amplification PCR targeting mitochondrial genome highly sensitive and specific in diagnosing malaria among returned travellers in Bergen, Norway. Malar J.

[28] Winskill P, Rowland M, Mtove G, Malima RC, Kirby MJ (2011). Malaria risk factors in north-east Tanzania. Malar J.

[29] Ishengoma DS, Mmbando BP, Segeja MD, Alifrangis M, Lemnge MM, Bygbjerg IC (2013). Declining burden of malaria over two decades in a rural community of Muheza district, north-eastern Tanzania. Malar J.

[30] Ishengoma DS, Mmbando BP, Mandara CI, Chiduo MG, Francis F, Timiza W (2018). Trends of *Plasmodium falciparum* prevalence in two communities of Muheza district North-eastern Tanzania: correlation between parasite prevalence, malaria interventions and rainfall in the context of re-emergence of malaria after two decades of progressive. Malar J.

[31] Shekalaghe SA, Bousema JT, Kunei KK, Lushino P, Masokoto A, Wolters LR (2007). Submicroscopic *Plasmodium falciparum* gametocyte carriage is common in an area of low and seasonal transmission in Tanzania. Trop Med Int Health.

[32] Schneider P, Bousema JT, Gouagna LC, Otieno S, Van De Vegte-Bolmer M, Omar SA (2007). Submicroscopic *Plasmodium falciparum* gametocyte densities frequently result in mosquito infection. Am J Trop Med Hyg.

[33] Okell LC, Bousema T, Griffin JT, Ouédraogo AL, Ghani AC, Drakeley CJ (2012). Factors determining the occurrence of submicroscopic malaria infections and their relevance for control. Nat Commun.

[34] Stewart L, Gosling R, Griffin J, Gesase S, Campo J, Hashim R (2009). Rapid assessment of malaria transmission using age-specific sero-conversion rates. PLoS ONE.

[35] WHO. World Malaria Report 2012. World Health Organization. 2013. p. 1–288. https://www.who.int/malaria/publications/world_malaria_report_2012/wmr2012_full_report.pdf. Accessed 12 June 2019.

[36] PMI. President’s Malaria Initiative: fighting malaria and saving lives: Tanzania country profile. USAID. 2016.

