# Supplementary figures and images for: Predictive markers of transmission in areas with different malaria endemicity in north-eastern Tanzania based on seroprevalence of antibodies against Plasmodium falciparum
Source: BMC Res Notes. 2021 Oct 30;14:404. doi: 10.1186/s13104-021-05818-y (PMC8557592; doi:10.1186/s13104-021-05818-y)

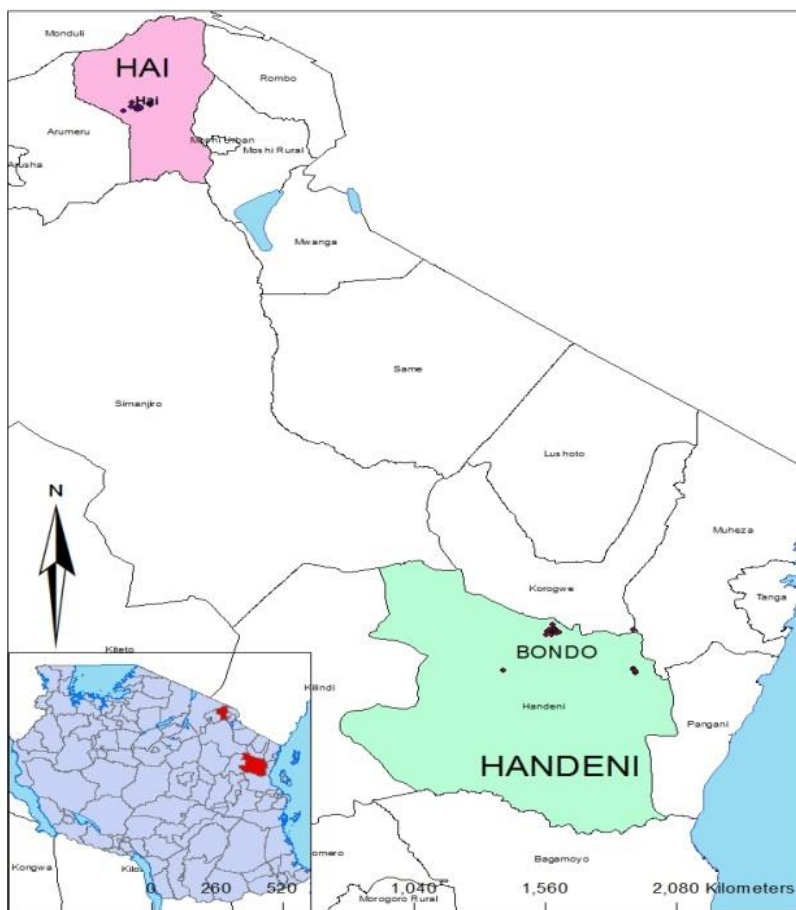

Supplement: Supplementary file 1 — Additional file 1: Figure S1. Map of Tanzania showing the study sites, the map was produced using ArcGIS version 10.3 software. [file 13104_2021_5818_MOESM1_ESM.pdf]

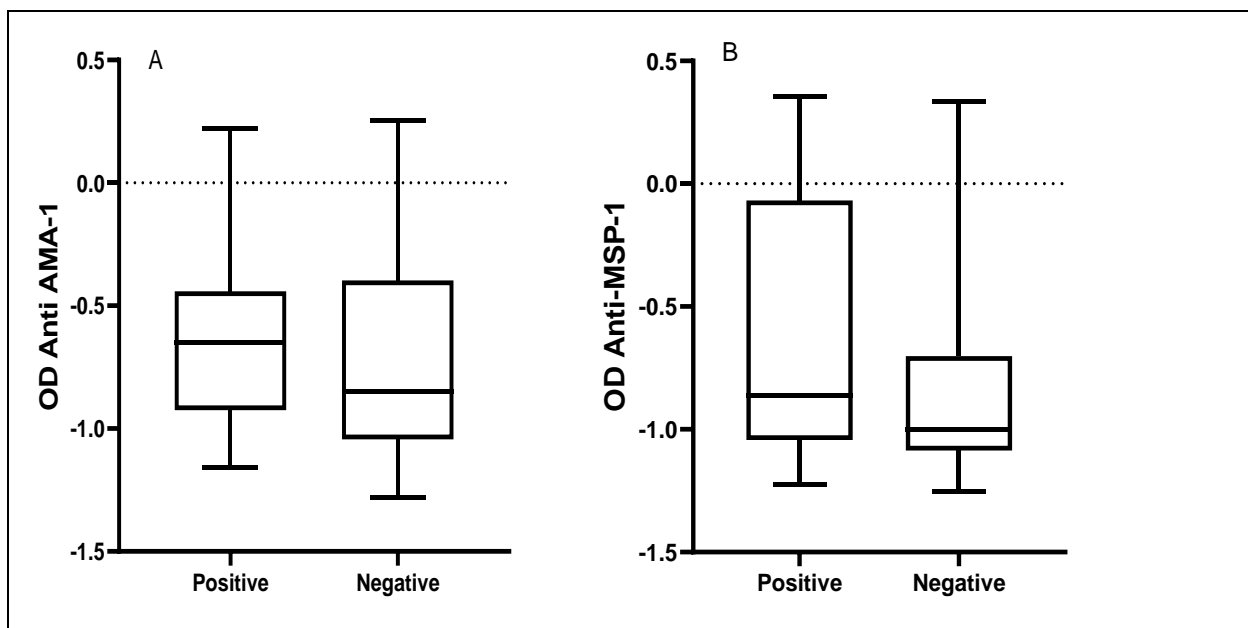

Supplement: Supplementary file 3 — Additional file 3: Figure S2. A graph showing mean OD values for PfAMA-1 (A) and PfMSP-119 (B) among malaria positive and negative individuals. Presented in the Y-axis is the Log10 transformed OD values among malaria positives and negatives (X-axis). [file 13104_2021_5818_MOESM3_ESM.pdf]
