# Supplementary material for: Predictive markers of transmission in areas with different malaria endemicity in north-eastern Tanzania based on seroprevalence of antibodies against Plasmodium falciparum
Source: BMC Res Notes. 2021 Oct 30;14:404. doi: 10.1186/s13104-021-05818-y (PMC8557592; doi:10.1186/s13104-021-05818-y)
Supplement: Supplementary file 2 — Additional file 2: Table S1. Prevalence of Malaria by serology, mRDT, Microscopy and PCR. [file 13104_2021_5818_MOESM2_ESM.pdf]

Table S1

		PfAMA-1 % (n)	*PfMSP-1*$_{19}$ % (n)	mRDT % (n)	PCR % (n)
Bondo	Positive	34.2 (188)	36.6 (201)	8.6 (47)	28.1 (154)
	Negative	65.8 (361)	348 (63.4)	91.4 (502)	71.9 (395)
Hai	Positive	13.8(33)	17.2 (41)	0.0 (0)	2.9 (7)
	Negative	86.2(206)	198 (82.8)	100.0 (239)	97.1 (232)
Total	Positive	28.0(221)	30.7 (242)	6.0 (47)	20.4 (161)
	Negative	72.0(567)	69.3 (546)	94.0 (741)	79.6 (627)
χ^2 p-value		χ^2=34.66 p<0.001	χ^2=29.62 p<0.001	*p<0.001	χ^2=64.64 p<0.001

*Computed by Fisher exact test.
